# Herpes zoster granulomatous dermatitis in metastatic lung cancer treated with nivolumab: A case report

**DOI:** 10.1111/1759-7714.13377

**Published:** 2020-03-05

**Authors:** Elisa Gozzi, Luigi Rossi, Francesco Angelini, Valentina Leoni, Patrizia Trenta, Giuseppe Cimino, Silverio Tomao

**Affiliations:** ^1^ UOC of Oncology ‐ ASL Latina‐ Distretto 1 University of Rome “Sapienza,” via Giustiniano snc – 04011 Aprilia Italy; ^2^ Medical Oncology Unit Regina Apostolorum Hospital Rome Italy; ^3^ Department of Medical Oncology Sapienza University of Rome, Medical and Surgical Sciences and Biotechnology Rome Italy; ^4^ Division of Medical Oncology A, Policlinico Umberto I Sapienza University of Rome Rome Italy; ^5^ Consorzio Interuniversitario per la Bio‐Oncologia (CINBO) Chieti Italy

**Keywords:** dermatologic adverse events, herpes zoster, immune checkpoint inhibitors, metastatic lung cancer, nivolumab

## Abstract

Granulomatous dermatitis (GD) is the most common among a variety of skin reactions that may occur in the varicella‐zoster virus (VZV) reactivation area. It is thought that the formation of granulomas may be the result of a delayed hypersensitivity reaction to viral envelope glycoproteins. Immune checkpoint inhibitors (ICIs), such as nivolumab stimulate T cells and promote hypersensitivity reactions, leading to the formation of granulomas in VZV wrapping proteins, thus triggering VZV‐GD. Few cases of the use of ICIs in patients diagnosed with VZV‐GD have been reported in the literature. Here, we report the clinical case of a patient with metastatic lung cancer which was treated with nivolumab who subsequently developed VZV‐GD. Accurate clinical diagnosis and prompt treatment with antiviral agents have resulted in a complete resolution of the clinical picture.

**Key points:**

**Significant findings**

- Treatment with ICIs may result in VZV reactivation.
- Accurate differential diagnosis and early treatment led to the resolution of VZV‐GD.

**What this study adds:**

- Few cases of ICI and VZV reactivation have been reported in the literature.
- Full and timely resolution of VZV‐GD allowed the continuation of ICI treatment.

## Introduction

Varicella‐zoster virus (VZV‐GD) is a cutaneous reaction that can appear in the area in which a reactivation of the VZV takes place. It may occur during treatment with ICIs but very few cases are described in the literature.^1^, ^2^

A differential diagnosis of dermatological adverse events (dAEs) related to treatment with ICIs should be carried out. dAEs occur in 34%–45% of patients treated with ICIs.^2^ They may present as a rash, pruritus, hypopigmentation/vitiligo, but also as xerosis, alopecia, stomatitis, urticaria, a photosensitivity reaction, hyperhidrosis and skin exfoliation.^3^ Management depends on classification of skin signs and symptoms and their severity.^2^

Here, we report the clinical case of a patient with metastatic lung cancer which was treated with nivolumab who subsequently developed VZV‐GD. Accurate clinical diagnosis and prompt treatment with antiviral agents have resulted in a complete resolution of the clinical picture.

## Case report

A 65‐year‐old woman presented to the clinic, following the appearance of supraclavicular lymphadenopathy with a diameter of 20 mm. A biopsy was performed, with a subsequent diagnosis of lung adenocarcinoma with mutation Exon 19 EGFR, ALK and ROS not rearranged, PDL‐1 negative.

Positron emission tomography (PET) and computed tomography (CT) scans were performed which indicated multiple mediastinal lymphadenopathy. Treatment with Afatinib was initiated and the disease was subsequently controlled for seven months. Following progression of the disease, no T790M mutation was detected in the circulating DNA, or after a new biopsy of the lesion.

The patient then commenced chemotherapy with six cycles of pemetrexed and cisplatin and whilst complete metabolic remission of a very short duration was achieved, it was followed by a rapidly evolving relapse. A PET/CT scan showed diffuse adenopathies, right adrenal loggia nodularity (SUV 12.1) right iliac adenopathies and cruralw inguin (SUV 13.7). The patient commenced treatment with nivolumab and achieved a complete response which was documented by PET scan. After six months of treatment, there was widespread erythema evident at the level of the humeral‐scapula articulation, with severe itching and pain. Subsequently, 2–3 days later, maculae and papules appeared which evolved into vesicles and then pustules. The area was affected throughout by severe itching and pain.

**Figure 1:**
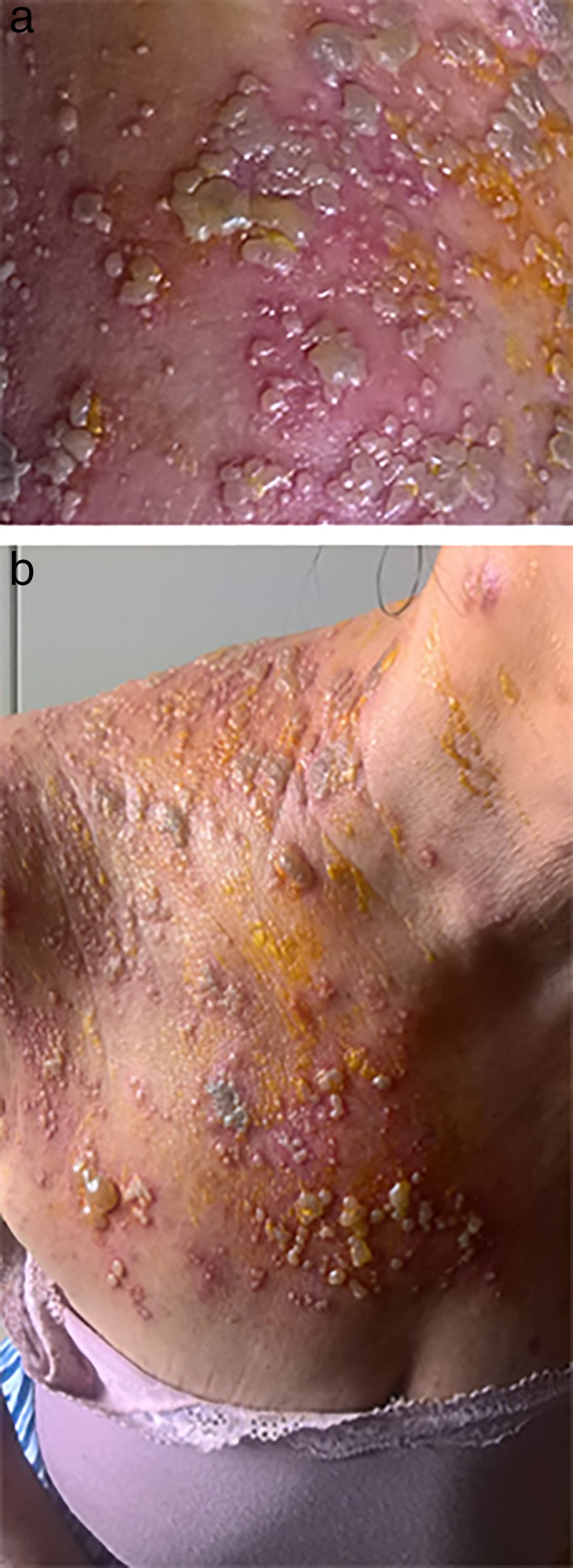
Herpes zoster infection with (**a**) necrotizing scapular and (**b**) supraclavicular cutaneous areas.

**Figure 2:**
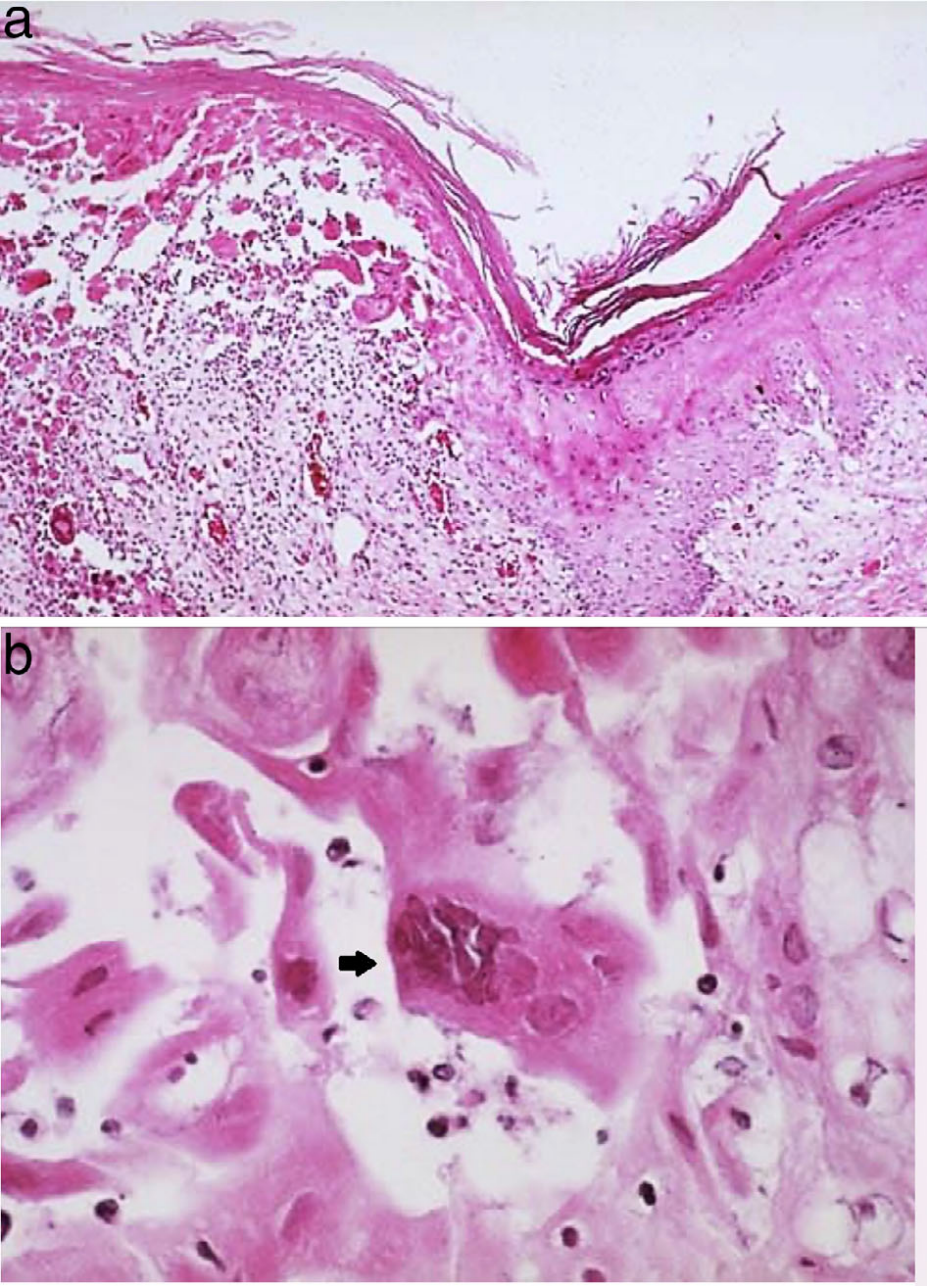
Histopathological analysis following skin biopsy. (**a**) High power intraepidermal vesicles with acantholysis indicative of herpesvirus infection (x200). (**b**) Swollen pale keratinocytes with enlarged slate‐grey nuclei and multinucleated cells (arrow) (x400).

Dermatological diagnosis was a grade 3 dAE due to VZV‐GD, with interesting scapular and supraclavicular cutaneous areas (Figs 1a and 1b). Histopathology of the skin biopsy confirmed it was VZV infection (Figs 2a and 2b). **T**reatment with nivolumab was subsequently temporarily discontinued. The patient commenced treatment with valaciclovir, 1000 mg three times a day for seven days in addition to fusidic acid cream which was applied twice a day to the damaged skin.

A clinical reassessment after three weeks from the diagnosis of the infection documented a good resolution of the clinical picture with improvement in the cutaneous erythema, the rash had dried with the formation of crusts and almost complete disappearance of the symptoms of itching.

At the time of writing this report, the patient is continuing treatment with nivolumab with excellent disease control.

## Discussion

The primary infection of VZV is chickenpox, manifested by viremia with a diffuse rash and seeding of multiple sensory ganglia where the virus establishes life‐long latency.^4^ The herpes zoster (HZ) virus is caused by the reactivation of latent VZV by the cranial nerve, or by ganglia of the dorsal root with spread of the virus along the sensory nerve to the dermatome. It is generally a mild, self‐limiting condition, but in some cases complications such as encephalitis, post‐herpetic neuralgia and Ramsay Hunt syndrome may occur, which can seriously affect the quality of life.^5^

There are more than one million cases of HZ in the United States each year with an annual rate of three to four cases per 1000 people. Most cases of HZ can be diagnosed clinically.^4^ Post VZV‐GD is the most frequent type within a variety of cutaneous reactions that can appear in the area in which a reactivation of this virus takes place.^1^, ^2^, ^3^, ^4^, ^5^, ^6^, ^7^ The formation of granulomas may be because of a delayed hypersensitivity reaction to viral envelope glycoproteins. These reactions have occurred in patients with lymphoproliferative syndromes, related in these cases to alterations in the number and function of immunoglobulins, cellular immunity and the increase in hypersensitivity reactions.^1^

Nivolumab is a human immunoglobulin monoclonal antibody, which binds to programmed death‐1 receptor (PD‐1) and blocks its interaction with the PD‐L1 and the PD‐L2. Interaction of the PD‐1 with the PD‐L1 and PD‐L2 ligands, which are expressed by the antigen‐presenting cells, cancer cell or by other cells in tumor microenvironment, involves inhibition of T cell proliferation and cytokine secretion. Nivolumab strengthens T cell responses, including antitumor responses.^6^ Some authors^1^ have postulated that nivolumab stimulated T cells, which promoted hypersensitivity reactions and the formation of granulomas to VZV envelope glycoproteins, triggering VZV‐GD.

Most frequent adverse events during treatment with ICIs are dAEs, which may range from vesicular, maculopapular, follicular, papular to exfoliative lesions or lupus‐like reactions.^2^

According to the European Society for Medical Oncology guidelines on the management of dAEs, the first requirement is to rule out any other etiology of the skin problem (Table 1). 8 In our case, accurate clinical diagnosis and prompt treatment resulted in a complete resolution of the clinical symptoms and the patient continues treatment with nivolumab with good disease control.

**Table 1:** ESMO guidelines on the management of dermatological adverse events (dAEs)

G	Macules/papules	Therapy
1	Covering <10% BSA	ICIs can be continued. TE, OA and TC
2	Covering 10%–30% BSA limiting ADL	ICIs can be continued. If not resolved, treatment should be interrupted until adverse skin event has reverted to grade 1. TE, OA and TC
3	Covering >30% BSA limiting selfcare ADL	Immediate interruption of ICIs, until these are back to grade 1. TE, OA and TC Systemic corticosteroids 0.5–1 mg/kg can be considered
4	Papulo‐pustular rash associated with life‐threatening superinfection Stevens‐Johnson syndrome TEN and bullous dermatitis covering >30% of BSA	Intensive care unit admission ICIs should be interrupted. Intravenous (methyl) prednisolone 1–2 mg/kg with tapering when toxicity resolves

ADL, activities of daily living; BSA, body surface area; G, guideline; ICIs, immune checkpoint inhibitors; OA, oral antihistamines; TC, topical corticosteroids; TE, topical emollients; TEN, toxic epidermal necrolysis.

## Disclosure

No authors report any conflict of interest.
